# Reweaving the Fabric of Mitochondrial Contact Sites in Astrocytes

**DOI:** 10.3389/fcell.2020.592651

**Published:** 2020-10-23

**Authors:** Matteo Bergami, Elisa Motori

**Affiliations:** ^1^Cologne Excellence Cluster on Cellular Stress Responses in Aging-Associated Diseases (CECAD), University Hospital Cologne, Cologne, Germany; ^2^Institute of Genetics, University of Cologne, Cologne, Germany; ^3^Center for Molecular Medicine Cologne, University of Cologne, Cologne, Germany; ^4^Max Planck Institute for Biology of Ageing, Cologne, Germany

**Keywords:** mitochondrial dynamics, endoplasmic reticulum, astrocytes, calcium, endfoot, Mfn2, mitochondria, MERCs

## Abstract

The endoplasmic reticulum (ER) and mitochondria are classically regarded as very dynamic organelles in cell lines. Their frequent morphological changes and repositioning underlie the transient generation of physical contact sites (so-called mitochondria-ER contacts, or MERCs) which are believed to support metabolic processes central for cellular signaling and function. The extent of regulation over these organelle dynamics has likely further achieved a higher level of complexity in polarized cells like neurons and astrocytes to match their elaborated geometries and specialized functions, thus ensuring the maintenance of MERCs at metabolically demanding locations far from the soma. Yet, live imaging of adult brain tissue has recently revealed that the true extent of mitochondrial dynamics in astrocytes is significantly lower than in cell culture settings. On one hand, this suggests that organelle dynamics in mature astroglia *in vivo* may be highly regulated and perhaps triggered only by defined physiological stimuli. On the other hand, this extent of control may greatly facilitate the stabilization of those MERCs required to maintain regionalized metabolic domains underlying key astrocytic functions. In this perspective, we review recent evidence suggesting that the resulting spatial distribution of mitochondria and ER in astrocytes *in vivo* may create the conditions for maintaining extensive MERCs within specialized territories – like perivascular endfeet – and discuss the possibility that their enrichment at these distal locations may facilitate specific forms of cellular plasticity relevant for physiology and disease.

## Introduction

**FIGURE 1:**
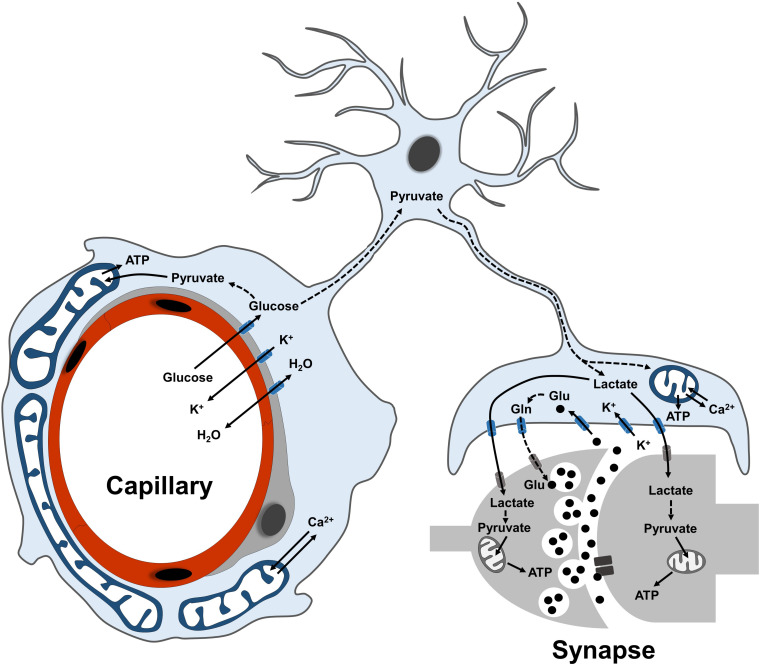
Overview of astrocyte functions in the neurovascular unit. Scheme illustrating the main metabolic functions of astrocytes at their perivascular (endfeet) and perisynaptic processes. Glucose is mostly taken up via the endfeet, where also ions and water molecules can be actively exchanged with the blood-brain barrier. Following glycolytic conversion to pyruvate, glucose can be utilized as energy substrate by astrocytic mitochondria or further converted into lactate to fuel synaptic transmission. At perisynaptic processes, astrocytes contribute to replenish the glutamate pool of neurotransmitters via the glutamate-glutamine cycle. Dashed lines between metabolites indicate a metabolic conversion. Plasma membrane transporters and ionic pumps are indicated with blue (for astrocytes) or gray (for neurons) symbols.

Substantial effort is being made in understanding the mechanisms that regulate tethering between mitochondria and other organelles, particularly the endoplasmic reticulum (ER), given that important functions have been ascribed to these mitochondria-ER contacts (MERCs) (Csordas et al., 2018). In particular, evidence exists for specific portions of the mitochondrial outer membrane being opposed by ER tubules within a distance of 15–30 nm. A growing number of tethering and regulatory proteins has been identified or proposed for maintaining in place these MERCs (Csordas et al., 2018). So far, these specialized domains have been implicated in the regulation of key cellular processes such as phospholipid metabolism (Vance, 2015; Dimmer and Rapaport, 2017), autophagosome formation (Hamasaki et al., 2013), and the transfer of Ca^2+^ between the two organelles (Csordas and Hajnoczky, 2009; Raffaello et al., 2016). Furthermore, MERCs also serve as sub-cellular signaling platforms, particularly in coordinating reactive oxygen species (ROS) signaling nanodomains (Booth et al., 2016). Finally, studies in cell lines have shown that the transient formation of MERCs is linked to membrane and organelle remodeling (Friedman et al., 2011; Lewis et al., 2016). While emerging evidence has begun disclosing the physiological and pathological relevance of MERCs in some peripheral tissues, our understanding of the principles regulating their formation and maintenance in the central nervous system, as well as their role for cellular function, is very limited. In part, this is likely due to the marked heterogeneity of cell sub-types characterizing brain tissue, which poses significant challenges in properly examining with sufficient spatial resolution the extent of MERCs and their dynamics *in situ* via imaging approaches. Electron microscopy is the method of choice for studying organelle contact sites and reconstructing organelle networks in whole cells, however, in brain tissue this approach may still be very time consuming on account of the geometric complexity of most cells contained within (e.g., neurons, astrocytes, and oligodendrocytes) and the intrinsic variability in cell sub-types across brain regions. As a result, these studies generally lead to the reconstruction of only few selected cells or even just part of them. Likewise, a systematic analysis of the signaling functions of MERCs in brain cells *in situ* may prove challenging to achieve. Yet, in parallel to recent studies that have begun addressing the extent of MERCs and potential regulatory tethering proteins in neurons (Hirabayashi et al., 2017; Wu et al., 2017), some of the implications of MERC dysfunction in neurodegeneration are also emerging (Area-Gomez and Schon, 2017; De Mario et al., 2017). Significant efforts are being also made to investigate organelle morphology, dynamics, and MERCs on at least one other type of brain cell, namely the astrocyte. Astrocytes exerts essential metabolic functions in the adult brain owing to their unique cellular architecture and positioning within the neurovascular unit (Figure 1), and recent studies have revealed an unexpected complexity of their mitochondrial and ER networks *in vivo*. Intriguingly, alongside with their elaborated morphologies, these two organelles were also found to be differentially distributed across cellular territories (Mathiisen et al., 2010; Cali et al., 2019; Göbel et al., 2020) and to give rise to a significant extent of MERCs in remote regions of the astrocyte – like the perivascular endfeet – where their specialized functions are most likely sustaining important roles in physiological and disease settings.

## Unexpected Complexity of Mitochondrial and ER Networks in Astrocytes

**FIGURE 2:**
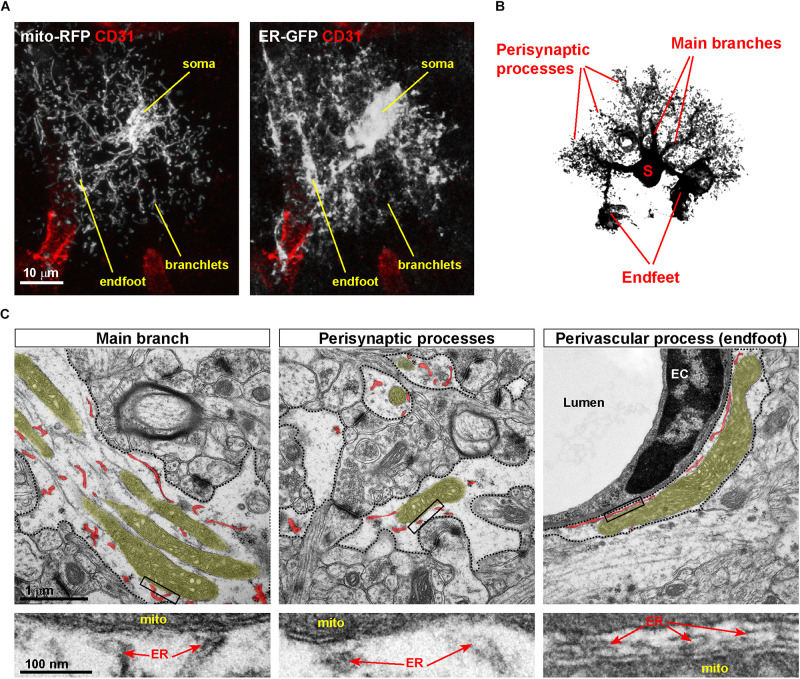
Asymmetric distribution of MERCs in astrocytic processes. **(A)** Example of a cortical astrocyte co-transduced with ER-GFP and mito-RFP viruses, showing the distribution of both organelles *in vivo* across astrocytic territories, particularly perivascular endfeet and fine branchlets. Immunostaining against the endothelial marker CD31, labeling the vasculature, is shown. **(B)** Scheme of an astrocyte showing the location of somas, main branches, perisynaptic, and perivascular processes (the latter ones visible as tube-like structures). **(C)** Electron microscopic pictures of distinct portions of the astrocyte (dashed areas correspond to astrocytic processes). While in main branches and endfeet large mitochondria are often visible, in processes surrounding synapses mitochondria display a smaller size. In each panel, mitochondria and ER are highlighted in different colors (yellow, mitochondria; red, ER). Lower panels depict zooms of the boxed areas, pointing to putative MERCs. EC, endothelial cell.

While the ER has been under intense investigation for its recognized role in Ca^2+^ handling in astrocytes (Bazargani and Attwell, 2016), the structure and function of astrocytic mitochondria have received much less attention. This underestimation of astrocytic mitochondrial metabolism has been, at least in part, a direct consequence of the generally accepted notion that astrocytes – in contrast to neurons – are mostly glycolytic in nature (Hertz et al., 2007; Supplie et al., 2017), and so this bias has for long time diverted the attention away from mitochondria, in which oxidative phosphorylation (OXPHOS) takes place. However, the recent employment of mitochondrial-targeted fluorescent indicators to investigate astrocytes *ex vivo* and *in vivo* disclosed a convoluted mitochondrial network, which is indicative of astrocytes relying substantially on this organelle for energy metabolism. In particular, the use of mito-YFP (and similar) reporters in astrocytes combined with high-resolution optic and electron microscopy recently allowed to fully appreciate the extent of mitochondrial mass and heterogeneity of mitochondrial morphologies displayed by astrocytes across their territories (Motori et al., 2013; Stephen et al., 2015; Agarwal et al., 2017; Gobel et al., 2018; Jackson and Robinson, 2018; Henneberger et al., 2019). While the exact morphological transformation of the mitochondrial network throughout astrocyte development still remains to be investigated, it is now clear that mature astrocytes possess a robust mitochondrial network, with large bundles of mitochondria that coalesce within main branches originating from the soma and invade the cell’s periphery (Figure 2A; Motori et al., 2013; Cali et al., 2019). Interestingly, the larger the distance from the soma, the smaller mitochondria appear with respect to their size, particularly within the numerous fine branches and branchlets that surround neuronal synapses (Figures 2B,C). This seemingly recapitulates what has been described in neurons, where active mechanisms sculpt mitochondrial morphology in distal dendrites and axons to achieve proper mitochondrial distribution at synapses (Li et al., 2004; Lewis et al., 2018). However, in contrast to fine astrocytic perisynaptic processes, which can at best accommodate the smallest mitochondria (Lovatt et al., 2007; Agarwal et al., 2017), perivascular endfeet (i.e., processes unsheathing most of the brain microvasculature and ensuring transfer of ions, water, and key metabolic substrates from/to the blood-brain-barrier) make a unique exception being capable to host quite elaborated morphologies, including long and branched mitochondria (Figure 2C; Mathiisen et al., 2010). Specifically, ultrastructural studies of the endfoot showed that the astrocytic terminals wrapping around microvessels are often packed with large mitochondria and, interestingly, ER tubules (Mathiisen et al., 2010; Göbel et al., 2020). Intriguingly, in contrast to the ER in perisynaptic processes which presents itself as short smooth tubules, the endfeet are characterized by a rather peculiar distribution of the ER. In these processes long bundles of both smooth and rough ER surround the basal lamina facing the endothelial side, thus generating a layer of ER membranes that virtually shield mitochondria from directly contacting the basal lamina (Göbel et al., 2020). While ER tubules are also found distributed across the entirety of the endfoot, the unique layered disposition of ER and mitochondria in the region adjacent the endothelium suggests that this arrangement of organelles may serve specific perivascular functions.

## Localized Enrichment of MERCs and its Consequence in Astrocytes

One of the best-studied aspects of astrocyte physiology is the remarkable extent and diversity of cytosolic Ca^2+^ transients displayed by the processes of these cells (Shigetomi et al., 2016). Thus, it is not surprising that these cells are differentially enriched in size and density of mitochondria as well as ER membranes across their territories, as both these two organelles play important roles in Ca^2+^ buffering and regulation. However, it is noteworthy that out of the vast number of mitochondria contained in all distal astrocytic processes, those confined within the ∼3 (in average) perivascular endfeet per astrocyte contain almost twice the extent of MERCs as compared to those in perisynaptic processes (Göbel et al., 2020). In part, this is facilitated by the natural enrichment in mitochondria and ER membranes within the endfoot. However, this asymmetry in the distribution of MERCs suggests that the perivascular region may be characterized by particularly elevated rates of lipid homeostasis, Ca^2+^ signaling and membrane dynamics, which so far represent the main functions ascribed to these contact sites (Csordas et al., 2018). Similar to other differentiated cell types, it is still unclear which exact proteins regulate the extent of MERCs in astrocytes out of the many possible proposed candidates (Csordas et al., 2018). While it is tempting to assume that many of the proposed natural tethers may share similar functions also in astrocytes, cell-type specificity within brain tissue may bring about additional layers of complexity, with certain tethers having for example a more prominent role in neurons than in astrocytes (Hirabayashi et al., 2017; Fecher et al., 2019). Furthermore, the intrinsic structural heterogeneity of MERCs among distinct astrocytic territories likely reflects a sub-specialization in ER-mitochondria tether proteins. If this is the case, one may expect an asymmetric enrichment in certain tether proteins among astrocytic territories, mirroring intracellular differences in organelle morphology and possibly function. Despite lack of clear evidence in astrocytes for the regulatory role of many previously proposed MERC-associated proteins (Csordas et al., 2018), recent work has begun to shed some light on the relevance of local MERC enrichment in astrocyte processes. For example, conditional deletion of the GTPase Mitofusin 2 (Mfn2), which resides at the ER-mitochondria interface (Hung et al., 2017) and has been proposed to regulate contact sites in cell lines (de Brito and Scorrano, 2008; Filadi et al., 2015; Naon et al., 2016), was indeed sufficient to alter the extent of MERCs *in vivo* and as a result interfere with the Ca^2+^ uptake capacity of astrocytic mitochondria (Göbel et al., 2020). In turn, the impaired mitochondrial Ca^2+^ buffering had direct consequences for local cytosolic transients, however, this effect appeared to be most prominent within the endfeet. Importantly, these functional alterations at the mitochondrial level were almost completely restored following expression of an artificial ER-mitochondria tether (Csordas et al., 2006; Göbel et al., 2020). This example demonstrates the functional relevance of an asymmetric distribution of MERCs in astrocytes, and provides a first evidence that a regionalized signaling in glial cells can be facilitated by local enrichments in mitochondrial and ER organelles. Yet, to which extent astrocytic MERCs can be considered as dynamic domains, especially in an *in vivo* situation, remains unclear. In cells *in vitro*, including astrocytes, these two organelles display a very active behavior, with mitochondria undergoing frequent fusion and fission events and ER tubules forming both stable and transient contacts at locations of future mitochondrial division (Friedman et al., 2011; Motori et al., 2013; Lewis et al., 2016). However, despite evidence for mitochondrial trafficking and fusion/fission dynamics in both acute and organotypic brain slice preparations (Motori et al., 2013; Jackson and Robinson, 2015; Stephen et al., 2015), distinct astrocytic territories appear to be independently regulated, with perivascular endfeet displaying much less dynamics as compared for instance to other branches and terminals (Göbel et al., 2020). However, it is unclear to what extent mitochondrial dynamics reflect actual changes in MERCs in astrocytes *in situ*. Given the spatial heterogeneity of some of the physiological functions to which MERCs may contribute (e.g., local Ca^2+^ dynamics) (Shigetomi et al., 2013; Agarwal et al., 2017; Bindocci et al., 2017), it is tempting to speculate that certain regions of the astrocyte might be subjected to a higher level of MERC regulation compared to other territories. For instance, bursts of dynamic changes in mitochondrial morphology and MERCs at perisynaptic processes may take place only in response to specific stimuli, as in the case of neurotransmitter spillover (Jackson et al., 2014; Stephen et al., 2015) or following induction of synaptic plasticity (i.e., potentiation) (Henneberger et al., 2019). Likely, the optimization of existing genetically encoded molecular sensors (Csordas et al., 2010) and the further development of *in situ* super-resolution approaches (Jakobs et al., 2020) may ultimately provide better access to live-cell organelle contact dynamics with minimal interference and possibly link these to specific astrocytic cellular functions (Iliff et al., 2012; Mishra et al., 2016).

## Relevance of MERCs for Astrocytic Reactivity States

In response to brain injury and inflammation, astrocytes are well known for their capability to acquire a so-called “reactivity state,” which is known to influence the progression of the initial insult (Khakh and Sofroniew, 2015). Indeed, reactive astrocytes – classically recognizable for their hypertrophic aspect and increased expression of markers such as glial fibrillary acidic protein (GFAP) (an intermediate filament marker) – have been identified in most neurological diseases (Sofroniew, 2014). Several studies, however, pointed out that this seemingly unique cellular state is rather characterized by a spectrum of heterogeneous changes, including profound alterations in gene and protein expression, thus suggesting the existence of multiple reactive states depending on type, severity, location and context of the triggering insult (Sofroniew, 2014; Liddelow and Barres, 2017). Further evidence now disclosed that reactive astrocytes may undergo significant metabolic rewiring when facing challenging conditions, as in the case of antiviral signaling response or in mouse models of Huntington disease (Chao et al., 2019; Polyzos et al., 2019). Yet, whether this rewiring under these conditions also involves changes in the metabolic functions of MERCs remains to be investigated. Metabolic flexibility in brain cells *in vivo* has been known for quite some time to be a fundamental feature of glial cells (Hertz et al., 2007; Weber and Barros, 2015), and while only recent work has begun to reveal neuron-specific forms of metabolic rewiring (Motori et al., 2020), the fact that astrocytes can efficiently reprogram their energy metabolism may explain their almost unique resilience to brain damage. In this respect, it is becoming clear that while reactive astrocytes can increase their glycolytic and glycogenolytic rates (Brown et al., 1995; Almeida et al., 2004; Motori et al., 2013), mitochondrial metabolism also plays a fundamental role in sustaining astrocyte functions following brain insult (Ignatenko et al., 2018; Fiebig et al., 2019). This reactivity state is indeed accompanied by a time-dependent transformation of the mitochondrial network in astrocytes directly exposed to acute injury and inflammation, which encompasses a general fragmentation shortly after injury followed by network re-tubulation during the next few weeks (Motori et al., 2013). Furthermore, simultaneous investigation of ER and mitochondrial network dynamics in injury-induced reactive astrocytes disclosed the marked accumulation of both these organelles in perivascular endfeet during an early phase after the initial insult, thus facilitating the formation of MERCs in these perivascular processes (Göbel et al., 2020). Preventing or enhancing this accumulation had effects not only on the magnitude and duration of local cytosolic Ca^2+^ transients of the astrocyte, but also influenced the extent of neo-angiogenesis in a model of penetrating brain injury (Göbel et al., 2020). While the precise mechanisms underlying this non-cell-autonomous effect on endothelial cells can only be speculated, these findings suggest that a dynamic reorganization of MERCs at precise locations of the astrocyte may serve to generate local metabolic domains important for tissue healing. Further studies are thus needed to investigate whether similar changes may also take place at perisynaptic processes. Likewise, additional work is necessary to establish the role of other tethers as well as putative regulatory MERC proteins other than Mfn2, and ascertain whether they might influence MERCs in astrocytes as well as astrocyte function. Still, these findings obviously raise the intriguing possibility that regulating the extent of MERCs in astrocytes (or other brain cells) may play a role in expressing distinct reactivity states, with direct consequences for neuronal viability (Anderson et al., 2016; Liddelow et al., 2017).

## Conclusion

Recent progress in microscopy and genetic techniques began unveiling an important role played by mitochondria and ER networks in regulating astrocytic functions, yet our understanding of organelle physiology and “contact-ology” in astrocytes is still rudimentary. For instance, we do not fully understand how much dynamic or static these contact sites are between ER and mitochondria. We also likely underestimate the extent to which MERCs differentially contribute to specific metabolic or signaling functions in distinct territories of the astrocyte. Likewise, very little is known about the role of astrocytic mitochondrial contacts with other organelles, for instance during postnatal astrocytic development, or following the acquisition of reactive states. In light of the recent suggestion that mitochondria may be transferred from/to astrocytes and other brain cells in settings of disease (Davis et al., 2014; Joshi et al., 2019), and that this transfer may even possibly compensate for certain metabolic deficits (Hayakawa et al., 2016), understanding the mechanisms regulating mitochondrial function at sites of contact with other organelles may lay the ground for targeted therapeutic approaches to improve brain repair during acute trauma and chronic neurodegeneration.

## Data Availability Statement

All datasets presented in this study are included in the article/supplementary material.

## Ethics Statement

The animal study was reviewed and approved by Landesamt für Natur, Umwelt und Verbraucherschutz Nordrhein-Westfalen.

## Author Contributions

All authors listed have made a substantial, direct and intellectual contribution to the work, and approved it for publication.

## Conflict of Interest

The authors declare that the research was conducted in the absence of any commercial or financial relationships that could be construed as a potential conflict of interest.

## References

[B1] Agarwal A. Wu P. H. Hughes E. G. Fukaya M. Tischfield M. A. Langseth A. J. (2017). Transient opening of the mitochondrial permeability transition pore induces microdomain calcium transients in astrocyte processes. *Neuron* 93 587–605e587. 10.1016/j.neuron.2016.12.034 28132831 PMC5308886

[B2] Almeida A. Moncada S. Bolanos J. P. (2004). Nitric oxide switches on glycolysis through the AMP protein kinase and 6-phosphofructo-2-kinase pathway. *Nat. Cell Biol.* 6 45–51. 10.1038/ncb1080 14688792

[B3] Anderson M. A. Burda J. E. Ren Y. Ao Y. O’shea T. M. Kawaguchi R. (2016). Astrocyte scar formation aids central nervous system axon regeneration. *Nature* 532 195–200. 10.1038/nature17623 27027288 PMC5243141

[B4] Area-Gomez E. Schon E. A. (2017). On the pathogenesis of alzheimer’s disease: the MAM hypothesis. *FASEB J.* 31 864–867. 10.1096/fj.201601309 28246299 PMC6191063

[B5] Bazargani N. Attwell D. (2016). Astrocyte calcium signaling: the third wave. *Nat. Neurosci.* 19 182–189. 10.1038/nn.4201 26814587

[B6] Bindocci E. Savtchouk I. Liaudet N. Becker D. Carriero G. Volterra A. (2017). Three-dimensional Ca2+ imaging advances understanding of astrocyte biology. *Science* 356:eaai8185. 10.1126/science.aai8185 28522470

[B7] Booth D. M. Enyedi B. Geiszt M. Varnai P. Hajnoczky G. (2016). Redox nanodomains are induced by and control calcium signaling at the ER-Mitochondrial interface. *Mol. Cell* 63 240–248. 10.1016/j.molcel.2016.05.040 27397688 PMC4998968

[B8] Brown G. C. Bolanos J. P. Heales S. J. Clark J. B. (1995). Nitric oxide produced by activated astrocytes rapidly and reversibly inhibits cellular respiration. *Neurosci. Lett.* 193 201–204. 10.1016/0304-3940(95)11703-Y 7478183

[B9] Cali C. Agus M. Kare K. Boges D. J. Lehvaslaiho H. Hadwiger M. (2019). 3D cellular reconstruction of cortical glia and parenchymal morphometric analysis from serial block-face electron microscopy of juvenile rat. *Prog. Neurobiol.* 183:101696. 10.1016/j.pneurobio.2019.101696 31550514

[B10] Chao C. C. Gutierrez-Vazquez C. Rothhammer V. Mayo L. Wheeler M. A. Tjon E. C. (2019). Metabolic control of astrocyte pathogenic activity via cPLA2-MAVS. *Cell* 179 1483–1498. 10.1016/j.cell.2019.11.016 31813625 PMC6936326

[B11] Csordas G. Hajnoczky G. (2009). SR/ER-mitochondrial local communication: calcium and ROS. *Biochim. Biophys. Acta* 1787 1352–1362. 10.1016/j.bbabio.2009.06.004 19527680 PMC2738971

[B12] Csordas G. Renken C. Varnai P. Walter L. Weaver D. Buttle K. F. (2006). Structural and functional features and significance of the physical linkage between ER and mitochondria. *J. Cell. Biol.* 174 915–921. 10.1083/jcb.200604016 16982799 PMC2064383

[B13] Csordas G. Varnai P. Golenar T. Roy S. Purkins G. Schneider T. G. (2010). Imaging interorganelle contacts and local calcium dynamics at the ER-mitochondrial interface. *Mol. Cell* 39 121–132. 10.1016/j.molcel.2010.06.029 20603080 PMC3178184

[B14] Csordas G. Weaver D. Hajnoczky G. (2018). Endoplasmic reticulum-mitochondrial contactology: structure and signaling functions. *Trends Cell Biol.* 28 523–540. 10.1016/j.tcb.2018.02.009 29588129 PMC6005738

[B15] Davis C. H. Kim K. Y. Bushong E. A. Mills E. A. Boassa D. Shih T. (2014). Transcellular degradation of axonal mitochondria. *Proc. Natl. Acad. Sci. U S A* 111 9633–9638. 10.1073/pnas.1404651111 24979790 PMC4084443

[B16] de Brito O. M. Scorrano L. (2008). Mitofusin 2 tethers endoplasmic reticulum to mitochondria. *Nature* 456 605–610. 10.1038/nature07534 19052620

[B17] De Mario A. Quintana-Cabrera R. Martinvalet D. Giacomello M. (2017). (Neuro)degenerated mitochondria-ER contacts. *Biochem. Biophys. Res. Commun.* 483 1096–1109. 10.1016/j.bbrc.2016.07.056 27416756

[B18] Dimmer K. S. Rapaport D. (2017). Mitochondrial contact sites as platforms for phospholipid exchange. *Biochim. Biophys. Acta. Mol. Cell Biol. Lipids* 1862 69–80. 10.1016/j.bbalip.2016.07.010 27477677

[B19] Fecher C. Trovo L. Muller S. A. Snaidero N. Wettmarshausen J. Heink S. (2019). Cell-type-specific profiling of brain mitochondria reveals functional and molecular diversity. *Nat. Neurosci.* 22 1731–1742. 10.1038/s41593-019-0479-z 31501572

[B20] Fiebig C. Keiner S. Ebert B. Schaffner I. Jagasia R. Lie D. C. (2019). Mitochondrial dysfunction in astrocytes impairs the generation of reactive astrocytes and enhances neuronal cell death in the cortex upon photothrombotic lesion. *Front. Mol. Neurosci.* 12:40. 10.3389/fnmol.2019.00040 30853890 PMC6395449

[B21] Filadi R. Greotti E. Turacchio G. Luini A. Pozzan T. Pizzo P. (2015). Mitofusin 2 ablation increases endoplasmic reticulum-mitochondria coupling. *Proc. Natl. Acad. Sci. U S A* 112 E2174–E2181. 10.1073/pnas.1504880112 25870285 PMC4418914

[B22] Friedman J. R. Lackner L. L. West M. Dibenedetto J. R. Nunnari J. Voeltz G. K. (2011). ER tubules mark sites of mitochondrial division. *Science* 334 358–362. 10.1126/science.1207385 21885730 PMC3366560

[B23] Göbel J. Engelhardt E. Pelzer P. Sakthivelu V. Jahn H. M. Jevtic M. (2020). Mitochondria-endoplasmic reticulum contacts in reactive astrocytes promote vascular remodeling. *Cell Metab.* 31 791–808. 10.1016/j.cmet.2020.03.005 32220306 PMC7139200

[B24] Gobel J. Motori E. Bergami M. (2018). Spatiotemporal control of mitochondrial network dynamics in astroglial cells. *Biochem. Biophys. Res. Commun.* 500 17–25. 10.1016/j.bbrc.2017.06.191 28676398

[B25] Hamasaki M. Furuta N. Matsuda A. Nezu A. Yamamoto A. Fujita N. (2013). Autophagosomes form at ER-mitochondria contact sites. *Nature* 495 389–393. 10.1038/nature11910 23455425

[B26] Hayakawa K. Esposito E. Wang X. Terasaki Y. Liu Y. Xing C. (2016). Transfer of mitochondria from astrocytes to neurons after stroke. *Nature* 535 551–555. 10.1038/nature18928 27466127 PMC4968589

[B27] Henneberger C. Bard L. Panatier A. Reynolds J. P. Medvedev N. I Minge D. (2019). LTP induction drives remodeling of astroglia to boost glutamate escape from synapses. *bioRxiv*

[B28] Hertz L. Peng L. Dienel G. A. (2007). Energy metabolism in astrocytes: high rate of oxidative metabolism and spatiotemporal dependence on glycolysis/glycogenolysis. *J. Cereb. Blood Flow Metab.* 27 219–249. 10.1038/sj.jcbfm.9600343 16835632

[B29] Hirabayashi Y. Kwon S. K. Paek H. Pernice W. M. Paul M. A. Lee J. (2017). ER-mitochondria tethering by PDZD8 regulates Ca(2+) dynamics in mammalian neurons. *Science* 358 623–630. 10.1126/science.aan6009 29097544 PMC5818999

[B30] Hung V. Lam S. S. Udeshi N. D. Svinkina T. Guzman G. Mootha V. K. (2017). Proteomic mapping of cytosol-facing outer mitochondrial and ER membranes in living human cells by proximity biotinylation. *Elife* 6:e24463 10.7554/eLife.24463.020 PMC5404927 28441135

[B31] Ignatenko O. Chilov D. Paetau I. De Miguel E. Jackson C. B. Capin G. (2018). Loss of mtDNA activates astrocytes and leads to spongiotic encephalopathy. *Nat. Commun.* 9:70. 10.1038/s41467-017-01859-9 29302033 PMC5754366

[B32] Iliff J. J. Wang M. Liao Y. Plogg B. A. Peng W. Gundersen G. A. (2012). A paravascular pathway facilitates CSF flow through the brain parenchyma and the clearance of interstitial solutes, including amyloid beta. *Sci. Transl. Med.* 4:147ra111. 10.1126/scitranslmed.3003748 22896675 PMC3551275

[B33] Jackson J. G. O’donnell J. C. Takano H. Coulter D. A. Robinson M. B. (2014). Neuronal activity and glutamate uptake decrease mitochondrial mobility in astrocytes and position mitochondria near glutamate transporters. *J. Neurosci.* 34 1613–1624. 10.1523/JNEUROSCI.3510-13.2014 24478345 PMC3905137

[B34] Jackson J. G. Robinson M. B. (2015). Reciprocal regulation of mitochondrial dynamics and calcium signaling in astrocyte processes. *J. Neurosci.* 35 15199–15213. 10.1523/JNEUROSCI.2049-15.2015 26558789 PMC4642244

[B35] Jackson J. G. Robinson M. B. (2018). Regulation of mitochondrial dynamics in astrocytes: mechanisms, consequences, and unknowns. *Glia* 66 1213–1234. 10.1002/glia.23252 29098734 PMC5904024

[B36] Jakobs S. Stephan T. Ilgen P. Bruser C. (2020). Light microscopy of mitochondria at the nanoscale. *Annu. Rev. Biophys.* 49 289–308. 10.1146/annurev-biophys-121219-081550 32092283 PMC7610798

[B37] Joshi A. U. Minhas P. S. Liddelow S. A. Haileselassie B. Andreasson K. I. Dorn G. W. II (2019). Fragmented mitochondria released from microglia trigger A1 astrocytic response and propagate inflammatory neurodegeneration. *Nat. Neurosci.* 22 1635–1648. 10.1038/s41593-019-0486-0 31551592 PMC6764589

[B38] Khakh B. S. Sofroniew M. V. (2015). Diversity of astrocyte functions and phenotypes in neural circuits. *Nat. Neurosci.* 18 942–952. 10.1038/nn.4043 26108722 PMC5258184

[B39] Lewis S. C. Uchiyama L. F. Nunnari J. (2016). ER-mitochondria contacts couple mtDNA synthesis with mitochondrial division in human cells. *Science* 353:aaf5549. 10.1126/science.aaf5549 27418514 PMC5554545

[B40] Lewis T. L. Jr. Kwon S. K. Lee A. Shaw R. Polleux F. (2018). MFF-dependent mitochondrial fission regulates presynaptic release and axon branching by limiting axonal mitochondria size. *Nat. Commun.* 9:5008. 10.1038/s41467-018-07416-2 30479337 PMC6258764

[B41] Li Z. Okamoto K. Hayashi Y. Sheng M. (2004). The importance of dendritic mitochondria in the morphogenesis and plasticity of spines and synapses. *Cell* 119 873–887. 10.1016/j.cell.2004.11.003 15607982

[B42] Liddelow S. A. Barres B. A. (2017). Reactive Astrocytes: Production, Function, and Therapeutic Potential. *Immunity* 46 957–967. 10.1016/j.immuni.2017.06.006 28636962

[B43] Liddelow S. A. Guttenplan K. A. Clarke L. E. Bennett F. C. Bohlen C. J. Schirmer L. (2017). Neurotoxic reactive astrocytes are induced by activated microglia. *Nature* 541 481–487. 10.1038/nature21029 28099414 PMC5404890

[B44] Lovatt D. Sonnewald U. Waagepetersen H. S. Schousboe A. He W. Lin J. H. (2007). The transcriptome and metabolic gene signature of protoplasmic astrocytes in the adult murine cortex. *J. Neurosci.* 27 12255–12266. 10.1523/JNEUROSCI.3404-07.2007 17989291 PMC6673251

[B45] Mathiisen T. M. Lehre K. P. Danbolt N. C. Ottersen O. P. (2010). The perivascular astroglial sheath provides a complete covering of the brain microvessels: an electron microscopic 3D reconstruction. *Glia* 58 1094–1103. 10.1002/glia.20990 20468051

[B46] Mishra A. Reynolds J. P. Chen Y. Gourine A. V. Rusakov D. A. Attwell D. (2016). Astrocytes mediate neurovascular signaling to capillary pericytes but not to arterioles. *Nat. Neurosci.* 19 1619–1627. 10.1038/nn.4428 27775719 PMC5131849

[B47] Motori E. Atanassov I. Kochan S. M. V. Folz-Donahue K. Sakthivelu V. Giavalisco P. (2020). Neuronal metabolic rewiring promotes resilience to neurodegeneration caused by mitochondrial dysfunction. *Sci. Adv.* 6:eaba8271. 10.1126/sciadv.aba8271 32923630 PMC7455195

[B48] Motori E. Puyal J. Toni N. Ghanem A. Angeloni C. Malaguti M. (2013). Inflammation-induced alteration of astrocyte mitochondrial dynamics requires autophagy for mitochondrial network maintenance. *Cell Metab.* 18 844–859. 10.1016/j.cmet.2013.11.005 24315370

[B49] Naon D. Zaninello M. Giacomello M. Varanita T. Grespi F. Lakshminaranayan S. (2016). Critical reappraisal confirms that Mitofusin 2 is an endoplasmic reticulum-mitochondria tether. *Proc. Natl. Acad. Sci. U S A* 113 11249–11254. 10.1073/pnas.1606786113 27647893 PMC5056088

[B50] Polyzos A. A. Lee D. Y. Datta R. Hauser M. Budworth H. Holt A. (2019). Metabolic reprogramming in astrocytes distinguishes region-specific neuronal susceptibility in huntington mice. *Cell. Metab.* 29 1258.e–1273.e. 10.1016/j.cmet.2019.03.004 30930170 PMC6583797

[B51] Raffaello A. Mammucari C. Gherardi G. Rizzuto R. (2016). Calcium at the center of cell signaling: interplay between endoplasmic reticulum, mitochondria, and lysosomes. *Trends Biochem. Sci.* 41 1035–1049. 10.1016/j.tibs.2016.09.001 27692849 PMC5123979

[B52] Shigetomi E. Bushong E. A. Haustein M. D. Tong X. Jackson-Weaver O. Kracun S. (2013). Imaging calcium microdomains within entire astrocyte territories and endfeet with GCaMPs expressed using adeno-associated viruses. *J. Gen. Physiol.* 141 633–647. 10.1085/jgp.201210949 23589582 PMC3639581

[B53] Shigetomi E. Patel S. Khakh B. S. (2016). Probing the complexities of astrocyte calcium signaling. *Trends Cell Biol.* 26 300–312. 10.1016/j.tcb.2016.01.003 26896246 PMC4946798

[B54] Sofroniew M. V. (2014). Astrogliosis. *Cold Spring Harb. Perspect. Biol.* 7:a020420. 10.1101/cshperspect.a020420 25380660 PMC4315924

[B55] Stephen T. L. Higgs N. F. Sheehan D. F. Al Awabdh S. Lopez-Domenech G. Arancibia-Carcamo I. L. (2015). Miro1 regulates activity-driven positioning of mitochondria within astrocytic processes apposed to synapses to regulate intracellular calcium signaling. *J. Neurosci.* 35 15996–16011. 10.1523/JNEUROSCI.2068-15.2015 26631479 PMC4666922

[B56] Supplie L. M. Duking T. Campbell G. Diaz F. Moraes C. T. Gotz M. (2017). Respiration-deficient astrocytes survive as glycolytic cells in vivo. *J. Neurosci.* 37 4231–4242. 10.1523/JNEUROSCI.0756-16.2017 28314814 PMC6596567

[B57] Vance J. E. (2015). Phospholipid synthesis and transport in mammalian cells. *Traffic* 16 1–18. 10.1111/tra.12230 25243850

[B58] Weber B. Barros L. F. (2015). The astrocyte: powerhouse and recycling center. *Cold Spring Harb. Perspect. Biol.* 7:a020396. 10.1101/cshperspect.a020396 25680832 PMC4665076

[B59] Wu Y. M. Whiteus C. Xu C. S. Hayworth K. J. Weinberg R. J. Hess H. F. (2017). Contacts between the endoplasmic reticulum and other membranes in neurons. *Proc. Nat. Acad. Sci. U S A* 114 E4859–E4867. 10.1073/pnas.1701078114 PMC5474793 28559323

